# Genomic Characterization of Phage ZP3 and Its Endolysin LysZP with Antimicrobial Potential against *Xanthomonas oryzae* pv. *oryzae*

**DOI:** 10.3390/v16091450

**Published:** 2024-09-11

**Authors:** Muchen Zhang, Xinyan Xu, Luqiong Lv, Jinyan Luo, Temoor Ahmed, Waleed A. A. Alsakkaf, Hayssam M. Ali, Ji’an Bi, Chengqi Yan, Chunyan Gu, Linfei Shou, Bin Li

**Affiliations:** 1 State Key Laboratory of Rice Biology and Breeding, Ministry of Agriculture Key Laboratory of Molecular Biology of Crop Pathogens and Insects, Zhejiang Key Laboratory of Biology and Ecological Regulation of Crop Pathogens and Insects, Institute of Biotechnology, Zhejiang University, Hangzhou 310058, China; 11816060@zju.edu.cn (M.Z.); 12016074@zju.edu.cn (X.X.); 22016087@zju.edu.cn (L.L.); temoorahmed@zju.edu.cn (T.A.); 2 Food Quality Supervision, Inspection and Testing Center of the Ministry of Agriculture and Rural Affairs (Shanghai), Shanghai Center of Agricultural Products Quality Safety, Shanghai 201708, China; 3 Department of Plant Quarantine, Shanghai Extension and Service Center of Agriculture Technology, Shanghai 201103, China; toyanzi@126.com; 4 Department of Life Sciences, Western Caspian University, Baku AZ1001, Azerbaijan; 5 MEU Research Unit, Middle East University, Amman 11192, Jordan; 6 Department of Botany and Microbiology, College of Science, King Saud University, Riyadh 11451, Saudi Arabia; walsakkaf.c@ksu.edu.sa (W.A.A.A.); hayhassan@ksu.edu.sa (H.M.A.); 7 Crop Institute, Ningbo Academy of Agricultural Sciences, Ningbo 315040, China; bihappy@foxmail.com (J.B.); yanchengqi@zaas.ac.cn (C.Y.); 8 Anhui Province Key Laboratory of Pesticide Resistance Management on Grain and Vegetable Pests, Institute of Plant Protection and Agro-Products Safety, Anhui Academy of Agricultural Sciences, Hefei 230031, China; 9 Station for the Plant Protection & Quarantine and Control of Agrochemicals Zhejiang Province, Hangzhou 310004, China

**Keywords:** *Xanthomonas oryzae* pv. *oryzae*, phage isolation, endolysin, biocontrol

## Abstract

*Xanthomonas oryzae* pv. *oryzae* (Xoo) is a significant bacterial pathogen responsible for outbreaks of bacterial leaf blight in rice, posing a major threat to rice cultivation worldwide. Effective management of this pathogen is crucial for ensuring rice yield and food security. In this study, we identified and characterized a novel Xoo phage, ZP3, isolated from diseased rice leaves in Zhejiang, China, which may offer new insights into biocontrol strategies against Xoo and contribute to the development of innovative approaches to combat bacterial leaf blight. Transmission electron microscopy indicated that ZP3 had a short, non-contractile tail. Genome sequencing and bioinformatic analysis showed that ZP3 had a double-stranded DNA genome with a length of 44,713 bp, a G + C content of 52.2%, and 59 predicted genes, which was similar to other OP1-type Xoo phages belonging to the genus *Xipdecavirus*. ZP3’s endolysin LysZP was further studied for its bacteriolytic action, and the *N*-terminal transmembrane domain of LysZP is suggested to be a signal–arrest–release sequence that mediates the translocation of LysZP to the periplasm. Our study contributes to the understanding of phage–Xoo interactions and suggests that phage ZP3 and its endolysin LysZP could be developed into biocontrol agents against this phytopathogen.

## 1. Introduction

Bacterial leaf blight (BLB), a devastating disease affecting rice, is induced by *Xanthomonas oryzae* pv. *oryzae* (Xoo), a Gram-negative bacterium responsible for significant global losses in both rice production and quality. In extreme scenarios, agricultural yield reductions can escalate to as much as 75%, with an annual impact on millions of hectares dedicated to rice cultivation [1,2]. Current strategies to mitigate BLB predominantly depend on conventional methods, encompassing physical interventions and chemical treatments, such as the removal of infected plant materials and the extensive use of pesticides [3]. However, such approaches frequently prove ineffective and engender significant concerns, including the evolution of drug-resistant bacterial strains and environmental pollution [4,5]. Consequently, the development of rice cultivars with enhanced resistance and the utilization of *Xanthomonas*-specific phages are increasingly regarded as more environmentally sustainable solutions.

Because of their unique lytic activity, phages—viruses that can infect and multiply in bacteria—are regarded as biocontrol agents against bacterial diseases [6]. Since the beginning of the twentieth century, *Xanthomonas* phages have been used as biocontrol agents [7]. For example, phage f20-Xaj controls walnut blight caused by *Xanthomonas arboricola* pv. *juglandis* [8], while phage Cp1 controls citrus canker caused by *Xanthomonas axonopodis* pv. *citri* [9]. In order to control BLB disease caused by Xoo, many phages have been reported in the past [10,11,12]. However, the existing phages are not effective enough to control all kinds of Xoo strains, and new phages to control Xoo are still required. Therefore, a novel Xoo phage, ZP3, which exhibits potent lytic activity against a variety of Xoo strains, was isolated and identified in this study. A thorough characterization was conducted, including analyses of morphology, host range, biophysical stability, and genomics.

Endolysins produced by phages can have a bacteriolytic action in vivo by cleaving the peptidoglycan layers of the bacterial cell wall [13]. During phage infection, the endolysin is delivered to the cell wall either via holin-dependent or holin-independent mechanisms. For most dsDNA phages, such as λ and T4, the endolysin relies on the holin, a small hydrophobic protein that inserts into the cytoplasmic membrane, to gain access to the cell wall and achieve bacterial lysis [14,15]. At the programmed time, the canonical holins form holes in the bacterial cell membrane, allowing the escape of the cytoplasmically located active endolysins into the periplasm through these holes and lysing the bacteria within seconds. However, some endolysins reach the cell wall in a holin-independent manner, such as the endolysins of the *Oenococcus oeni* phage fOg44 and the *Escherichia coli* phage P1. Lys-44 of fOg44 possesses a cleavable signal sequence at the *N*-terminus. Consequently, the catalytic domain of Lys-44 is exported by the *sec* translocon, and its signal sequence is subsequently cleaved by leader peptidase. However, phage fOg44 has a canonical holin gene [16]. Endolysins of phage P1 can be exported using signal–arrest–release (SAR) sequences to interact with the host secretion system. The Lyz SAR sequence is both necessary and sufficient for *sec*-mediated export to a membrane-tethered state, followed by release into the periplasm. Cellular lysis induced by Lyz results from the slow, spontaneous release of a minor portion of membrane-anchored Lyz. The activation of the holin at the programmed lysis time facilitates the rapid and complete release of the SAR endolysin from the membrane. This phenomenon is attributed to the dissipation of the proton motive force (pmf) subsequent to holin activation [17].

Endolysins, which retain specific bactericidal activity, can be utilized to control bacterial disease [18,19]. In fact, endolysins have been applied in various fields, including the control of human and agricultural bacterial diseases [20]. Given the advantages of phages and their endolysins’ applications, in this work we show that the newly discovered phage ZP3 could serve as a biocontrol agent. Its endolysin is also investigated and is shown to exhibit bacteriolytic action.

## 2. Materials and Methods

### 2.1. Bacterial Strains, Plasmids, and Culture Conditions

Among the 55 strains of rice bacterial blight utilized in this experiment, 54 strains were isolated and identified from the main areas where rice bacterial blight has a high incidence in Guangdong, Zhejiang, Yunnan, Guangxi, Henan, and Fujian. The other strains were preserved in the laboratory. Bacterial strains were grown at 30 °C in nutrient agar (NA) broth (glucose 2.5 g/L, beef extract 3 g/L, NaCl 5 g/L, and tryptone 10 g/L). *E. coli* strains were cultured at 37 °C in Luria–Bertani (LB) agar or broth medium (yeast extract 5 g/L, tryptone 10 g/L, and NaCl 10 g/L). The plasmids utilized in this work are listed in Table 1. If necessary, 50 μg/mL kanamycin, 100 μg/mL ampicillin, or 1 mM IPTG (isopropyl-β-D-thiogalactoside) was supplemented to the corresponding culture medium [21].

### 2.2. Isolation, Purification, and Concentration of Phage

Xoo strains from different regions were selected as potential host bacteria, and the phage was isolated afterwards. Briefly, the diseased rice leaves from Zhejiang were twice cleaned with sterile water after being immersed in 75% alcohol for 30 s for disinfection. Afterward, the diseased leaves were cut and soaked in sterile water, then shaken at 30 °C and 200 rpm for 1 h. The phages were obtained by means of filtration and sterilization through a PES membrane filter (0.22 μm, Merck Millipore Ltd., Cork, Ireland), and their lytic ability was proven via the double-layer agar and bacterial growth curve method [10]. Purified phages with uniformly sized plaques were picked, stored in SM buffer, and propagated according to the phage lysate method [22]. Phage stock was mixed with fresh host Xoo bacterial culture and incubated at 30 °C for 30 min before shaking at 30 °C and 200 rpm until the bacterial culture became clear. We combined DNase I and RNase A at a final concentration of 1 mg/mL with the cell lysate, mixing and incubating this mixture for 1 h at 37 °C. Then, 0.584 g/L NaCl was added, and the whole mixture was centrifuged at 4 °C and 11,000× *g* for 10 min after being incubated at 4 °C for 1 h. Solid polyethylene glycol 8000 (PEG8000) was added to the supernatant at a final concentration of 10% (*w*/*v*) and stored at 4 °C overnight. After 10 min of centrifugation at 4 °C and 11,000× *g*, phage pellets were collected and resuspended in SM buffer. Chloroform of the same volume was added, and the mixture was vortexed for 30 s. Concentrated phage particles were obtained by collecting the supernatant after centrifuging the mixture at 4 °C and 3000× *g* for 15 min.

### 2.3. Optimal Multiplicity of Infection (MOI) Determination

The MOI is the ratio of phages to bacteria at the time of infection [23]. Briefly, 1 × 10^9^ PFU/mL adjusted phage stock was 10-fold diluted to 1 × 10^4^ PFU/mL with SM buffer. Each phage dilution was mixed and incubated with the host Xoo strain (1 × 10^7^ CFU/mL) with 1:1 (*v*/*v*) at 30 °C for 30 min before shaking for 6 h and centrifugation at 11,000× *g* for 10 min. The supernatant, which contained the propagated phage stock, was tested for the phage titer. The best MOI was the phage–bacteria ratio that multiplied to the maximum phage titer.

### 2.4. Determination of Phage Adsorption Rate

Host Xoo strain G5 in NA broth was incubated at 30 °C after being infected by the phage at the optimal MOI. Samples were taken at 0 min, 1 min, 2 min, 3 min, 4 min, 5 min, 10 min, 15 min, 20 min, 25 min, and 30 min from the phage–bacteria combinations and centrifuged at 11,000× *g* for 10 min. After passing the supernatants through a 0.22 μm PES membrane filter, the double-layer agar technique was used to measure the titer of unadsorbed free phages present in the supernatant. The experiment was repeated three times independently.

### 2.5. Determination of Phage One Step Growth Curve

All the phage samples were filtered before being utilized in every experiment to exclude the possibility of contamination. The phage was incubated with the Xoo strain at an optimal MOI of 30 °C for 5 min for adsorption to avoid non-synchronous infection. The unadsorbed phages by the host bacteria in the supernatant were removed after centrifugation at 13,000× *g* for 30 s. Then, 5 mL of NA medium was used to resuspend the pellet before shaking at 30 °C and 200 rpm. A volume of 100 μL of the mixture was tested every 10 min to determine the phage titer. We took the infection time as the abscissa and the logarithm of the phage titer as the ordinate to draw a phage one-step growth curve [24].

### 2.6. Transmission Electron Microscopic (TEM) Observation

Phage ZP3 was visualized by means of the negative staining method of TEM. In brief, 2% (*w*/*v*) phosphotungstic acid (pH 6.8) was used to negatively strain 10 μL of purified phage particles on a copper grid coated with carbon. A JEM-1230 transmission electron microscope (JEOL, Tokyo, Japan) was used to observe the phage particles. A total of 15 phage particles were used to calculate the head and tail length of ZP3. Bacteria TEM observation was conducted, followed by the previous protocol [25]. *E. coli* BL21(DE3) cells containing pET28a-LysZP, pET28a-HolZP, pET28a-LysZP+HolZP, pET28a-LysZPΔTMD, and empty pET28a vectors were inducted with IPTG at 16 °C and 180 rpm for 6 h and centrifuged at 5000× *g* for 5 min, and then were washed 3 times with 0.1 M phosphate-buffered saline (PBS) solution. Then, 2.5% (*v*/*v*) glutaraldehyde was used to fix the bacterial samples overnight. After that, 1% (*w*/*v*) osmium tetroxide in 0.1 M PBS was added to the agarose embedded-bacterial samples for 1 h followed by three washes with 0.1 M PBS. Samples were dehydrated for 15 min using graded ethanol (30%, 50%, 70%, and 80%) and acetone (90%, 95%, and 100%), and then were embedded in a low-viscosity embedding resin Epon 812. After being sliced in a LEICA EM UC7 ultrathin microtome, bacterial samples were observed under a Hitachi H-7650 microscope (Tokyo, Japan).

### 2.7. Determination of the Biophysical Stability of Phage ZP3

To determine its thermal stability, phage ZP3 at 1 × 10^9^ PFU/mL was treated at different temperatures (4 °C, 20 °C, 40 °C, 60 °C, and 80 °C) for different lengths of time (30 min, 60 min, and 120 min), and then the titer of the phage was detected using double-layer agar plates. Each temperature treatment was performed in triplicate. To determine its pH stability, phage ZP3 was diluted to a final titer of 1 × 10^9^ PFU/mL by buffers with different pH values (1–14) and incubated for 30 min, 60 min, and 120 min before the titer measurement. To determine its disinfectant stability, phage ZP3 was diluted to a final titer of 1 × 10^9^ PFU/mL with ethanol or isopropanol, the most commonly used alcohol disinfectants [26], at 30% and 60% concentrations and incubated for 30 min, 60 min, and 120 min before taking the titer measurement. Each treatment was repeated three times. We used the different treatments as the x-axis, while the y-axis showed the viability, indicating the ability to form plaques on a host lawn.

### 2.8. Determination of Phage Host Range

The host range of phage ZP3 was determined through the spot-test method [27]. The bacterial strains were prepared to the same concentration of 1 × 10^7^ CFU/mL before mixing with molted semi-solid NA medium to form the double-layer agar plates. Then, 2 μL of phage ZP3 (1 × 10^9^ PFU/mL) was spotted on the plate surface. After overnight incubation at 30 °C, the host range was recorded through the plaque formation. Tested strains were freshly isolated in the laboratory or obtained from the bacterial strain collection of the laboratory. The genome accession numbers of some of the strains were as follows: Pxo99A (GCA_000019585.2); ScYc-b (GCA_002895725.2); OS198 (GCA_003382875.1); YN1 (GCA_002895745.1); YN7 (GCA_002895765.1); YN11 (GCA_002895775.1); YN18 (GCA_002895805.1); YN24 (GCA_002895825.1); HEN11 (GCA_002895685.1); GD414 (GCA_002895675.1); FuJ (GCA_002895665.1).

### 2.9. Phage DNA Extraction, Genome Sequencing, and Bioinformatic Analysis

Phage DNA was extracted through implementing the extraction kit from Beijing BLKW Biotechnology Co., Ltd. (Beijing, China) following the protocol. Briefly, the supernatant after centrifugation of the phage lysis culture was first digested with RNase A and DNase I to remove the residual DNA and RNA of the host bacteria. Thereafter, SDS was added, and the residual fragments were removed via centrifugation. Phage DNA in the lysate supernatant was transferred to the given adsorption column, and the impurities of phage DNA, such as salt, cell metabolites, and proteins, were removed through a series of rapid rinsing centrifugation steps. Phage DNA was obtained through eluent [28]. The whole genome was sequenced in Biozeron (Shanghai, China) using the high-throughput sequencing method, which involves random fragmentation of DNA followed by end repair and adapter ligation. Specifically, Illumina TruSeq™ Nano DNA Sample Prep Kit (San Diego, CA, USA) was used to construct the library, starting with 1 μg of DNA. The DNA was fragmented to 300–500 bp using Covaris M220 sonication, followed by end repair, A-tailing at the 3′ end, and adapter ligation. Trimmomatic version 0.39 was used to evaluate the reading quality. The paired-end reads were spliced with multiple Kmer parameters using ABySS version 2.1.5 (http://www.bcgsc.ca/platform/bioinfo/software/abyss, accessed on 27 December 2022) splicing software to obtain the optimal assembly results. The GapCloser version 1.12 (https://sourceforge.net/projects/soapdenovo2/files/GapCloser/, accessed on 27 December 2022) software was used to perform local joining of the gaps and base correction on the assembly results. Default parameters were used in these pieces of software. The assembled phage genome was cyclized using a circulator (http://sanger-pathogens.github.io/circlator/, accessed on 27 December 2022). Also, the origin and orientation of the genome were adjusted according to a highly identical phage genome from NCBI alignment version 2.13.0. The potential genes were predicted by GeneMarkS version 1.14_1.25_lic (http://topaz.gatech.edu/GeneMark/genemarks.cgi, accessed on 26 April 2023) and the RAST server (http://rast.nmpdr.org/rast.cgi, accessed on 26 April 2023). The protein function was annotated in the non-redundant protein database using BLASTp version 2.13.0 (http://blast.ncbi.nlm.nih.gov/, accessed on 26 April 2023). The tRNA-encoding genes were predicted by tRNA scan-SE (https://lowelab.ucsc.edu/tRNAscan-SE/, accessed on 26 April 2023). The representation of phage ZP3’s genome was generated using CGView Server version 1.36 (http://stothard.afns.ualberta.ca/cgview_server/, accessed on 6 July 2024) [29]. Phage genome termini and the detection of terminal repeat sequences of phage ZP3 were predicted by the PhageTerm program version 1.0.12 [30]. A neighbor joining tree was drawn using MEGA 7.0. TMHMM Server v. 2.0 (https://services.healthtech.dtu.dk/service.php?TMHMM-2.0, accessed on 26 April 2023) and Signal P 4.1 Server (https://services.healthtech.dtu.dk/service.php?SignalP-4.1, accessed on 26 April 2023). The biological characteristics and 3D structure of LysZP and HolZP proteins were predicted by SWISS-MODEL (https://swissmodel.expasy.org/, accessed on 26 April 2023). Conservative domain analysis was conducted through BLASTp in NCBI (https://blast.ncbi.nlm.nih.gov/Blast.cgi, accessed on 8 September 2024). Sequence alignment was conducted by DNAMAN version 6, published in 2005 by Lynnon Corporation (http://www.lynnon.com, accessed on 26 April 2023).

### 2.10. Recombinant Plasmids Construction

To construct the recombinant plasmid pET28a-LysZP, the Lys gene of ZP3 was amplified through PCR using the ZP3 genome and primer pairs Lys-F/R (Table 2). LysZP DNA fragments were cloned into BamHI-cleaved pET28a empty plasmids through homologous recombination using the ClonExpress II One Step Cloning Kit (Vazyme Biotechnology Co., Ltd., Nanjing, China) in accordance with the manufacturer’s instructions. The recombinant plasmids were heat-shock transformed into *E. coli* BL21 (DE3) for protein expression. pET28a-HolZP, pET28a-LysZP+HolZP, and pET28a-LysZPΔTMD were constructed using the same protocol [31]. The other three holin candidates (ORF25, 28 and one potential holin gene located at 22,907–23,200 bp) were also connected with the pET28a vector, respectively.

### 2.11. Growth Measurement

Through the comparison of the bacterial numbers at OD_600_, the influence of LysZP and HolZP on bacterial growth was determined. *E. coli* BL21(DE3) cells containing pET28a-LysZP, pET28a-HolZP, pET28a-LysZP+HolZP, pET28a-LysZPΔTMD, and an empty pET28a vector were cultured to OD600 = 0.8. The above bacterial culture was transferred to LB liquid broth and cultured to OD_600_ = 0.3. Afterwards, each bacterial culture received a final dose of 0.5 mM IPTG and was incubated at 16 °C and 180 rpm for 0–7 h. The OD_600_ value was measured every hour after incubation for 7 h. The protocol of growth measurement for the three other holin candidates was the same as HolZP.

### 2.12. Staining of Live/Dead Cells and Flow Cytometry Monitoring

The living conditions of *E. coli* cells expressing different proteins 6 h after IPTG induction were tested using a fluorescent backlight bacterial viability kit from Invitrogen. Under the inverted confocal microscope LSM780 (Carl Zeiss Microscopy GmbH, Munich, Germany), live cells showed green fluorescence with SYTO 9, while dead cells were red with propidium iodide [32]. For flow cytometry observation, bacterial cells with different treatments were stained with 50 mg/L propidium iodide in the dark for 15 min and washed twice with 0.1 M PBS before being observed using a FACSVerse cytometer (BD Biosciences, San Jose, CA, USA) [33].

### 2.13. β-Galactosidase Activity Assay

For *E. coli* cells expressing different proteins after IPTG induction at 37 °C for 0.5 h, 3 h, and 6 h, the supernatant was obtained after centrifugation at 12,000× *g* rpm for 5 min. Then, 500 μL of the supernatant was fully mixed with 100 μL of 20 mM o-nitrophenyl-β-galactoside (ONPG) at 45 °C for 30 min. After this, 600 μL of 0.5 M Na_2_CO_3_ was added to the mixture to stop the color reaction. The OD_420_ nm value was measured to determine the β-galactosidase activity through a microplate spectrophotometer (Thermo Fisher Scientific Inc., Waltham, MA, USA).

### 2.14. Protein Expression and Antibacterial Effect Assay

*E. coli* cells carrying pET28a-LysZP and empty pET28a as a negative control were incubated in LB (with 50 µg/mL kanamycin) at 37 °C and 200 rpm until OD_600_ = 0.6. After 20 h of induction at 16 °C and 180 rpm with 0.5 mM IPTG, the bacterial pellets were collected through centrifugation at 4 °C and 5000× *g* for 15 min, resuspended in 0.1 M PBS, and lysed by sonication for 10 min (400 W ultrasound for 3 s, interval of 2 s). Following the centrifugation of the bacterial debris at 9000× *g* for 20 min at 4 °C, the lysate supernatant was collected and filtered through a PES membrane filter (0.45 μm, Merck Millipore Ltd., County Cork, Ireland). We dripped 10 μL of the target protein onto the Xoo-based double-layer agar plate and cultured at 37 °C for 24 h. The antibacterial effect was tested through measuring the width of the inhibition zone.

## 3. Results

### 3.1. Isolation and Morphology of the Xoo Phage ZP3

**Figure 1:**
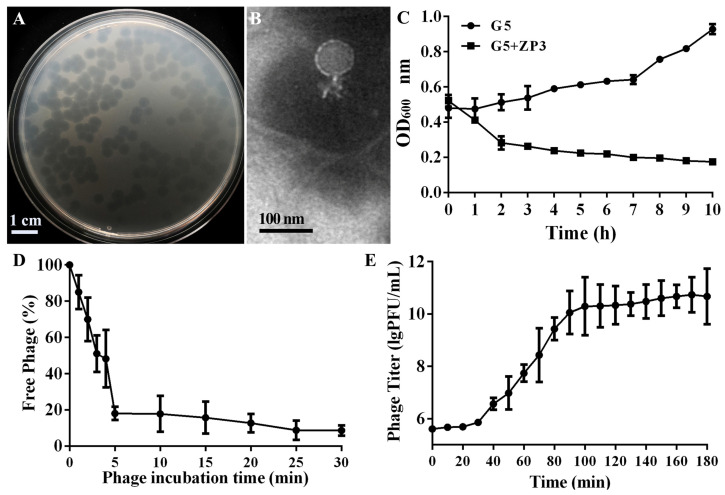
Characteristics of phage ZP3. (**A**) Phage plaques of ZP3 infecting Xoo strain G5 in double-layer agar plates. (**B**) Transmission electron microscopy image of ZP3. (**C**) Bacterial growth curve of Xoo strain G5 with and without phage ZP3. (**D**) Adsorption rate of phage ZP3. (**E**) One-step growth curve of ZP3.

Using Xoo strain G5 as the host, a novel phage named ZP3 was isolated from rice leaves in Zhejiang, China. Phage ZP3 formed clear, uniformly sized plaques of 3.7 ± 0.4 mm diameter (Figure 1A). Phage ZP3 was further propagated and purified for morphological visualization. TEM observation showed that ZP3 had an isometric head (64.12 ± 9.03 nm) with a short non-contractile tail (21.64 ± 5.70 nm) (Figure 1B). When phage ZP3 was added to Xoo_G5 culture, the OD_600_ dropped from 0.52 to 0.28 during 2 h and continued to decrease until it reached 0.17 after 10 h. The bacterial culture became clear, and lysed bacterial debris could be seen in the culture, thus proving that ZP3 is a lytic phage (Figure 1C). ZP3 had a high rate of adsorption of more than 80% within 5 min of incubation. After 30 min of incubation, there were only 8.7% free phages in the supernatant (Figure 1D). A one-step growth curve was created after the incubation of ZP3 and the Xoo strain at 30 °C at 0.01 MOI to evaluate the infection dynamics. The results indicate that the latent duration of phage ZP3 was 30 min, having a large burst size of 626 ± 215 plaque-forming units (PFUs) per infected cell (Figure 1E).

### 3.2. Host Range of Phage ZP3

In order to evaluate the biological control potential of ZP3, its lytic activity against 56 Xoo strains was assessed via the spot-test method. Fifty-five Xoo strains were obtained from the main rice-growing regions in China (19 strains from Guangdong Province, 14 from Zhejiang Province, 8 from Liaoning Province, 5 from Guangxi Province, 5 from Yunnan Province, 2 from Henan Province, 1 from Sichuan Province, and 1 from Fujian Province), along with 1 standard Xoo strain, Pxo99A. One standard *Xanthomonas oryzae* pv. *oryzicola* (Xoc) strain RS105 and three other bacterial strains isolated from similar rice paddy areas, including two *Acidovorax oryzae* (Ao) strains and one *Burkholderia seminalis* (Bs) strain, were also assessed. The results show that ZP3 could only infect Xoo strains, suggesting infection specificity towards this species. ZP3 had lytic action on 28 out of the 56 Xoo tested strains, suggesting a relatively broad spectrum of activity within Xoo strains (Table 3, Figure S1).

### 3.3. Biophysical Stability of Phage ZP3

**Figure 2:**
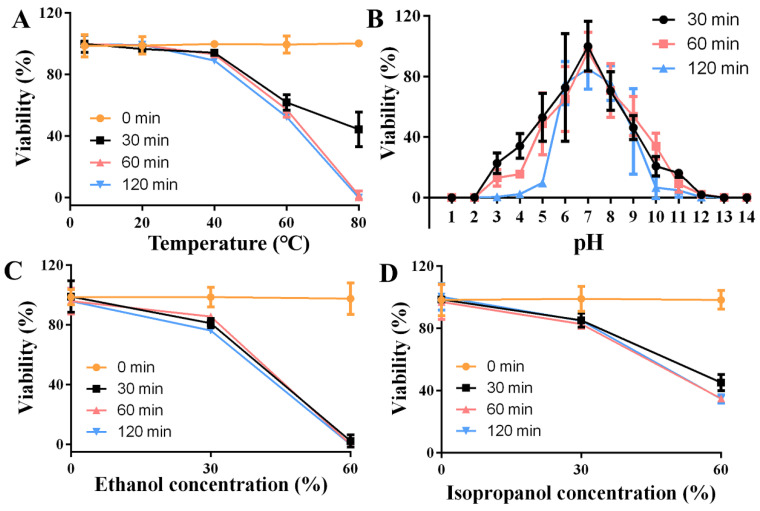
Biophysical stability of phage ZP3. (**A**) The temperature stability of ZP3. (**B**) The pH stability of ZP3. The ethanol (**C**) and isopropanol (**D**) stability of ZP3.

The stability of bacteriophages in different environments and their ability to reproduce are important properties of phages. Therefore, we evaluated the stability of ZP3 under different temperatures, under different pH conditions, and under the pressure of different common disinfectants. We treated 1 × 10^9^ PFU phages at 4 °C, 20 °C, 40 °C, 60 °C, and 80 °C for 30 min, 60 min, and 120 min, respectively. The results show that a low temperature or room temperature has little effect on ZP3 stability. Overall, 93–100% of phages were viable at 4–40 °C, while when the temperature exceeded 60 °C, the phage titer decreased rapidly, with the phage viability decreasing from 62% to 44% after 30 min of treatment under 60–80 °C. Notably, with the increase in time, the titer decreased further, and the phage viability dropped to 0% after 120 min of treatment at 80 °C, indicating that ZP3 is unstable at high temperatures (60–80 °C) but is stable at lower temperatures (4–40 °C) (Figure 2A). Additionally, the stability of ZP3 to various pH values was examined. As shown in Figure 2B, when phage ZP3 was treated with various pH values for 30 min, 60 min, and 120 min, the highest titer was detected at pH = 7, which was considered the optimal pH for ZP3. Two common disinfectants, ethanol and isopropanol, were selected to treat ZP3 for 30–120 min (Figure 2C,D). When the ethanol and isopropanol concentration was 30%, ZP3’s viability after 120 min decreased to 76% and 85%, respectively. When the concentration increased to 60%, almost no phages survived in ethanol, while 47% of phages survived in isopropanol after 30 min, which showed that ZP3 was more sensitive to ethanol treatment.

### 3.4. ZP3 Genome Sequencing, Annotation, and Phylogenetic Analysis

**Figure 3:**
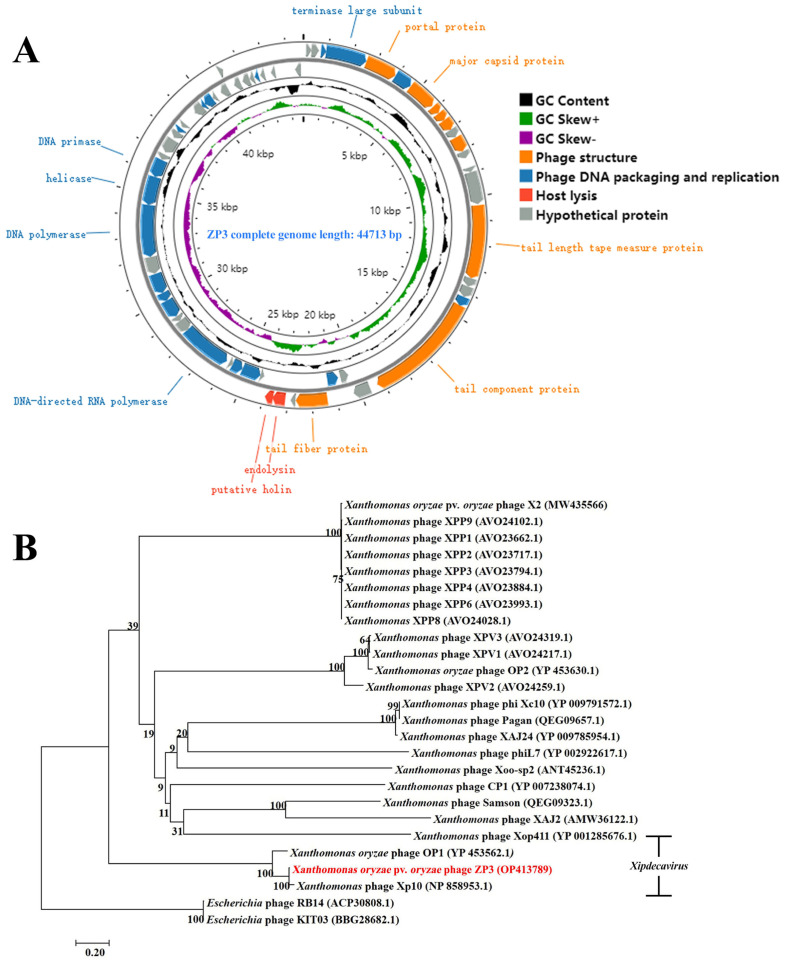
(**A**) Phage ZP3’s annotated genome map. Arrows are used to denote the predicted genes, and each arrow’s orientation indicates the transcription direction. Genes in the three functional modules are represented in orange (phage structure), blue (phage DNA packaging and replication), red (host lysis), and gray (hypothetical protein). (**B**) ZP3 phage neighbor-joining phylogenetic tree analysis based on terminase large subunit amino acid sequences; 1000 replications of the bootstrap values. Xoo phages belonging to the genus *Xipdecavirus* are marked.

To explore the link between ZP3 and other reported *Xanthomonas* phages, ZP3 genomic DNA was extracted, sequenced, assembled, and annotated (GenBank accession number OP413789). According to the results, phage ZP3 had a linear double-stranded DNA (dsDNA) genome with a length of 44,713 bp and a G + C content of 52.2%, which was lower than that of its host Xoo_G5 (63.4%) (Figure 3A). PhageTerm report indicates that ZP3 adopts a COS (3′) packaging mode, consistent with the packaging mode of HK97 phage (accession number: NC_002167.1), making it a cos phage with a 3′ overhang, generating fixed DNA ends with cohesive ends that possess a 3′ overhang, and no direct terminal repeats were detected. Genome alignment of ZP3 to the sequences of the GenBank database was performed via BLASTp, which showed that ZP3 had significant similarity only to Xoo phages, namely *Xanthomonas oryzae* phage OP1 (accession number: NC_007709.1, 87% coverage and 84.95% identity), Xp10 (accession number: NC_004902.1, 85% coverage and 86.23% identity), and Xop411 (accession number: NC_009543.1, 82% coverage and 88.99% identity), thus representing its novelty and the enrichment of Xoo phages. According to new taxonomic guidelines, ZP3 was identified as a member of the *Xipdecavirus* genus based on its close genome homology to *Xanthomonas* phage OP1 [34].

Fifty-nine protein-encoding ORFs in total were identified in the phage ZP3 genome using the GeneMarkS and RAST servers; 33 ORFs were predicted in the minus strand, while the remaining ORFs were predicted to be in the plus strand (Table S1). Furthermore, of the 59 protein-coding genes predicted in the genome, 29 were annotated with known functions, and no tRNA-encoding genes were detected. According to bioinformatic analysis, the ZP3 genome has three functional modules: the phage structure, phage DNA packaging and replication, and host lysis. Eight gene products were related to phage structure function, including tail fiber, tail component protein, head-tail connector protein, and capsid protein, while nineteen proteins were involved in the phage DNA packaging and replication. The DNA packaging module contained the genes encoding the terminase large subunit (ORF4) and phage peptidase (ORF49). The replication-related genes encode DNA polymerase, DNA primase/helicase, DNA binding protein, DNA-binding protein, and other related proteins. The presence of endolysin (ORF26) related to host lysis was consistent with the lytic ability of phage ZP3. In addition, some proteins are necessary for host lysis, such as exonucleases (encoded by ORF 35 and 37), endonucleases (encoded by ORF 36), and an inhibitor of transcription initiation (encoded by ORF 45). Phages can also interfere with the host’s RNA polymerase (RNAP) and other transcriptional activators and/or regulators, thereby disrupting normal host–cell functions and contributing to the process of host lysis [35]. While these proteins do not directly cause lysis, they play important roles in the phage life cycle that can lead to the eventual lysis of the host–cell. The exonucleases and endonuclease can degrade the host’s DNA, while the inhibitor of transcription initiation can shut down host gene expression, both of which are steps that can contribute to the final stage of lysis. Lysogeny-associated genes, or genes encoding virulence factors, antibiotic resistance, or toxins, were not detected in the ZP3 genome, indicating its safety for use as a biocontrol agent.

In order to examine the connection between phage ZP3 and other *Xanthomonas* phages, the phylogenetic evolutionary process was analyzed at the protein level based on the sequence of the terminase large subunit, which is conserved in tailed phages. The results reveal that phage ZP3 can be grouped into the OP1-type Xoo phages and is closely related to *Xanthomonas* phage Xp10 (Accession NP858953.1), while it is phylogenetically distant from other *Xanthomonas* phages (Figure 3B).

### 3.5. In Silico Identification of Phage ZP3 Endolysin

**Figure 4:**
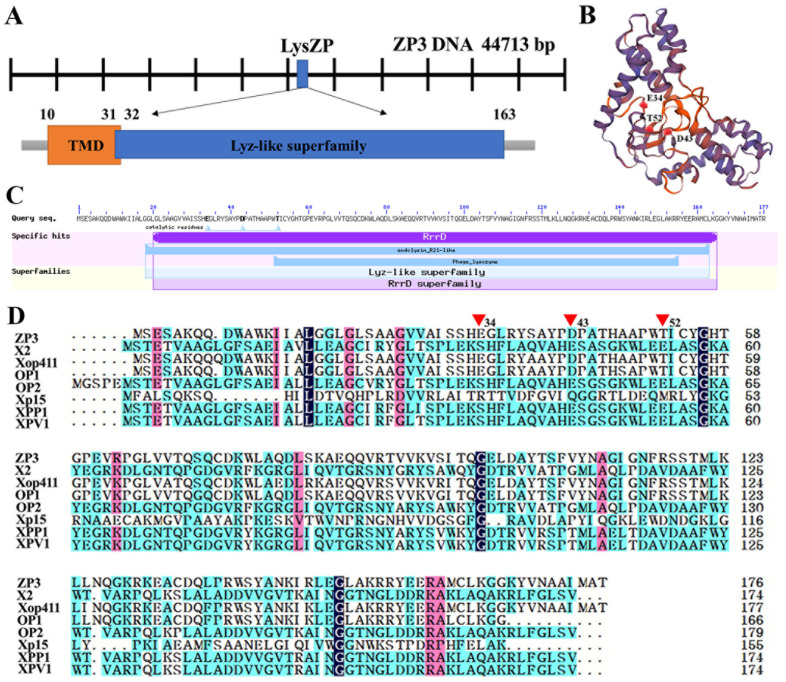
In silico characterization of the ZP3 endolysin. (**A**) Genomic organization and schematic representation of LysZP. Amino acid position is indicated by numbers. (**B**) Three-dimensional structure prediction of LysZP. (**C**) Annotation of LysZP protein. (**D**) Sequence alignment of LysZP with various Xoo phage endolysins, including X2 (MW435566), Xop411 (ABK00175.1), OP1 (YP_453585.1), OP2 (YP_453642.1), Xp15 (YP_239293.1), XPP1 (YP_010052413.1), and XPV1 (AVO24202.1). Red triangles (E34, D43, and T52) represent the catalytic triad residues. Different colors of the letters mean the homology level (black: 100%; pink: ≥ 75%; blue: ≥ 50%).

Endolysin is a lytic enzyme that is ubiquitous in dsDNA phages and is used to degrade the bacteria cell wall’s peptidoglycan, inducing bacterial lysis. After genome sequencing and annotation of ZP3, ORF26 (23,100–23,633 bp) was predicted to encode phage endolysin, referred to as LysZP. Endolysin LysZP was composed of 177 amino acids with a molecular mass of 19.5 kDa. Bioinformatic analysis revealed that LysZP has no typical signal peptide but one transmembrane domain (TMD) located between residues 10 to 32, whose *N*-terminal region is on the outside while its C-terminus is on the inside. A lysozyme-like domain, a member of the Lyz-like superfamily containing the conserved catalytic triad (E34, D43, and T52), was identified within the residues 31–163 of LysZP by Protein BLAST (BLASTp) in NCBI (Figure 4).

### 3.6. Transmembrane Domain Is the Key to Cell Lysis Induced by Endolysin

To characterize the function of LysZP, pET28a-LysZP was constructed and expressed in *E. coli* BL21 (DE3). The amino acid residues 10–32 in LysZP represent a TMD region and a hydrophobic domain, considered to be a possible endolysin SAR domain [17]. However, initial genome analysis by RAST and Blastp did not identify the ZP3 holin successfully. Since the holin-encoding genes are generally located in the vicinity of the endolysin-encoding genes with a highly charged C-terminal region and contained at least one TMD, we analyzed all the possible genes (ORF25, 27, 28, and one potential holin gene located at 22,907–23,200 bp). Among all the four holin candidates, ORF27 (HolZP), a small protein with 111 amino acids (23,602–23,937 bp) and a molecular mass of 12.2 kDa, possessed a TMD between the amino acid residues 10–32, which was considered to be a putative type III holin [36].

**Figure 5:**
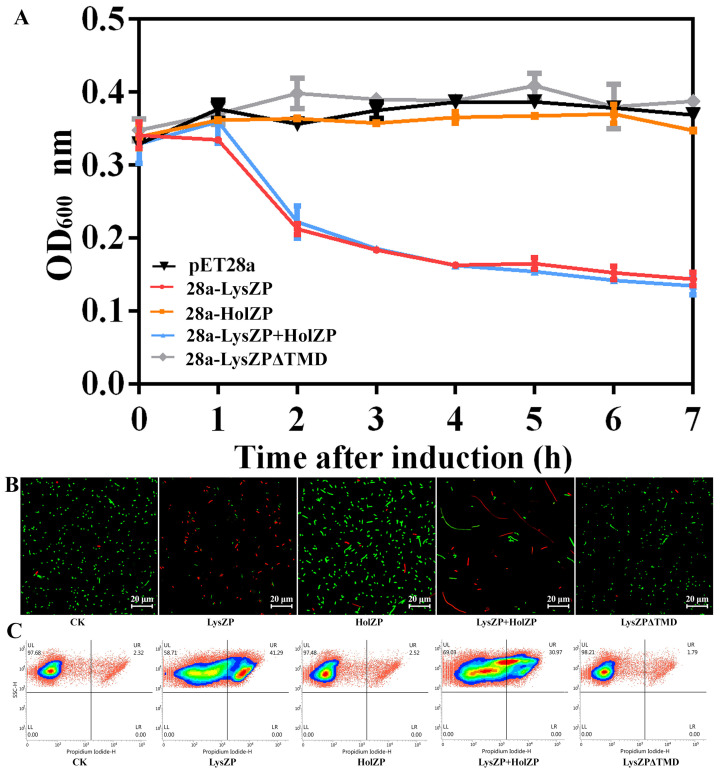
Effects of LysZP and HolZP on bacterial growth. (**A**) Growth curves of *E. coli* expressing LysZP, HolZP, LysZP+HolZP, and LysZPΔTMD after IPTG induction. (**B**) Live and dead bacterial fluorescent staining experiment (green for live cells and red for dead cells). (**C**) Flow cytometry scatter plots.

We therefore constructed plasmids pET28a-HolZP, pET28a-LysZP+HolZP, and pET28a-LysZPΔTMD by deleting the TMD and connecting the ninth amino acid with the thirty-third amino acid directly (Figure S2). When LysZP was expressed alone, cell lysis occurred 2 h after IPTG induction, and the OD_600_ value of bacteria dropped from 0.34 to 0.21 and decreased continuously to 0.14 after 7 h of induction with clear and viscous bacterial fluid, thus showing the deleterious effects on bacteria cells. The same situation was observed when co-expressing LysZP and HolZP together. The bacteria’s OD_600_ of 0.33 at the beginning decreased to 0.13 after IPTG induction for 7 h, which showed that HolZP had few effects on LysZP. In addition, the expression of HolZP alone had no effects on the bacterial growth. Since the expression of LysZP alone played a role in cell lysis without the help of HolZP, we further explored the key factor that contributed to LysZP function. As shown in Figure 5A, the truncated protein of LysZP lost its lysis function, and the OD_600_ increased from 0.34 to 0.39 after 7 h of induction, thus showing a similar trend to the negative control (*E. coli* BL21 containing the empty vector pET28a), whose OD_600_ increased from 0.33 to 0.37.

To further validate that the expression of LysZP caused cell death, live-dead bacterial staining and flow cytometry of PI-stained dead cells were performed (Figure 5B). For the negative control, the majority of the bacteria showed green fluorescence 6 h post IPTG induction, indicating that the bacteria were alive. This result was consistent with that of the flow cytometry, in which 97.68% cells were alive while 2.32% cells were dead (Figure 5C). In contrast, *E. coli* expressing LysZP had a high ratio of dead cells (41.29%), and most of the cells were red, representing dead cells, which means that the expression of LysZP disrupted the integrity of the membrane, killing the cells. For HolZP, most cells were green and only 2.52% were dead, which was consistent with the bacterial growth curve.

When TMD was deleted from LysZP, the expression of LysZPΔTMD had no effect on bacterial viability, with only 1.79% dead cells. The majority of the cells were green, which means that LysZP relies on TMD to exert its lytic effect on cells. This result is consistent with a previous study on the endolysin of *Acidovorax oryzae* phage AP1, whose TMD acted as an SAR sequence, inducing the transportation of endolysins to the periplasm [32]. SAR sequences usually have a high Gly/Ala content (40–60%) and zero to two basic residues [17]. Similarly, LysZP had a TMD region in aa positions 10–32, among which 39% of the amino acid residues were Ala plus Gly and one Lys residue. Combined with the fact that the expression of LysZP alone can lead to bacterial lysis, it is speculated that the TMD region of LysZP could function as an SAR sequence.

### 3.7. Expression of Endolysin LysZP Affects Membrane Integrity

**Figure 6:**
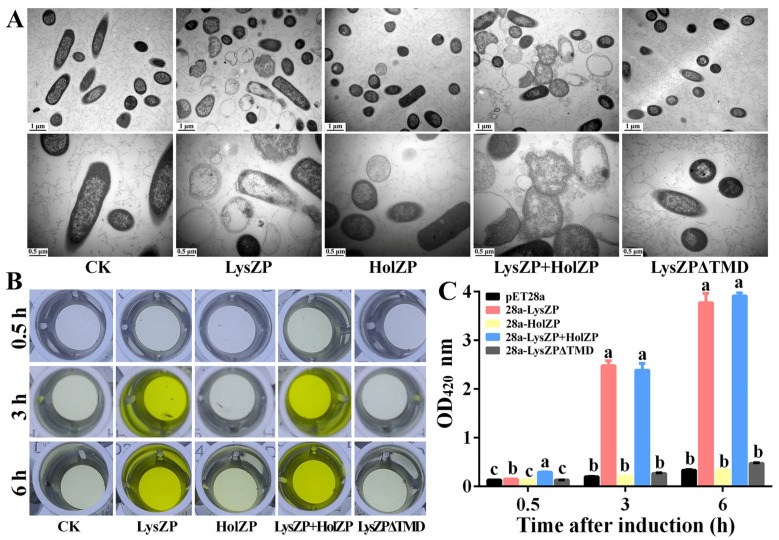
Effects of lysZP and HolZP on cell membrane integrity. (**A**) Morphological changes in bacterial cells under TEM. (**B**,**C**) β-galactosidase activity after IPTG induction for 0.5 h, 3 h, and 6 h. Color change in plates (**B**) and the corresponding OD_420_ values (**C**). Columns with different letters (a–c) are significantly different according to the LSD test (*p* < 0.05).

Previous experiments showed that the expression of LysZP significantly affects bacterial growth and viability. We further observed the microstructure of bacterial cells 6 h after IPTG induction under a transmission electron microscope (TEM). As shown in Figure 6A, cells of the negative control (*E. coli* BL21) had an intact cell wall and membrane, and the cell color was dark with a high density of cellular contents. However, for cells expressing LysZP alone or co-expressing LysZP with HolZP, most of them were broken and the cell membrane shrank dramatically. The irregular cell wall structure led to the leakage of cellular contents. The cell wall and membrane were separated, empty, and swollen, and fragmented cells were observed, thus representing the severe damage caused by LysZP to cell integrity. In comparison, most of the *E. coli* cells expressing HolZP remained intact, while some of them shrank slightly and the cell color became lighter. Similarly, LysZPΔTMD expression led to a reduction in cell lysis ability, and the cells were integrated with dense cellular contents.

To quantify the degree of cell fragmentation and the compromising of the cell membrane’s integrity, β-galactosidase activity was tested. β-galactosidase is a cytosolic enzyme that cannot be detected extracellularly unless the cell envelope is broken. β-galactosidase activity of *E. coli* cells expressing various vectors was detected 0.5 h, 3 h, and 6 h after IPTG induction under OD_420_ nm. Consistent with the previous experiments, HolZP and LysZPΔTMD displayed no significant difference compared with the negative control during the whole induction process. The OD_420_ value increased slowly from 0.13 (0.5 h) to 0.45 (6 h), indicating an intact cell membrane. Therefore, β-galactosidase cannot break the barrier to be detected. In comparison with cells expressing LysZP alone, the OD_420_ value increased drastically, ranging from 0.15 after 0.5 h of induction to more than 3.77 at 6 h of induction, with the color changing from transparent to dark yellow. This indicated that the expression of LysZP destroyed the structural integrity of the cell membrane and induced the leakage of intracellular β-galactosidase. This color change was more evident in cells co-expressing LysZP with HolZP: with an OD_420_ value of 0.30 at 0.5 h, they displayed a light-yellow color, while when the OD_420_ increased drastically to 3.92, they displayed a dark yellow color. The OD_420_ values of LysZP alone and LysZP with HolZP were significantly higher than those of the other three treatments during the whole induction process (Figure 6B). The results of the β-galactosidase tests clearly clarify the effect of LysZP on cell membrane integrity, which was the main reason for cell lysis and the leakage of cellular contents.

### 3.8. Antibacterial Effect of ZP3 Endolysin on Xoo

**Figure 7:**
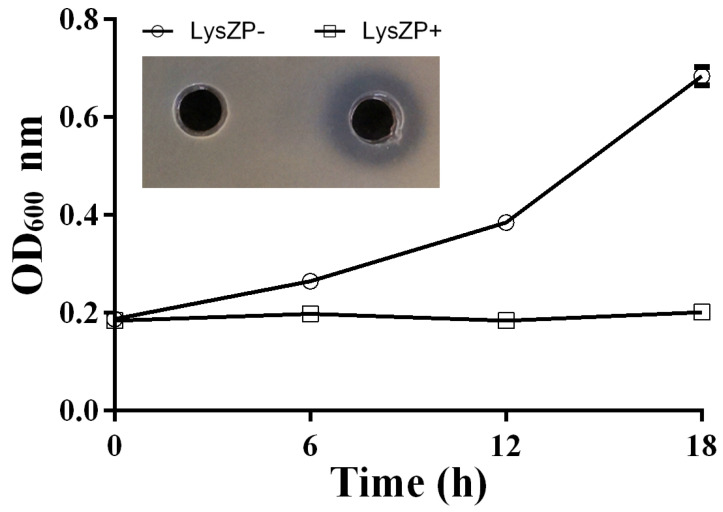
Bacteriolysis effect of LysZP on Xoo_C2R. Xoo_C2R growth was measured with and without LysZP protein treatment. The three replicates’ standard deviations are shown by the error bars. LysZP against Xoo_C2R was tested through measuring the width of the inhibition zone.

As mentioned above, LysZP expression led to *E. coli* cell lysis. On this basis, the expression of LysZP protein was examined to test its antibacterial effect on Xoo cells. The results show that LysZP could inhibit the growth of all the tested Xoo strains, even strains with resistance to phage ZP3, as illustrated for the strain Xoo_C2R (Figure 7) [25]. *E. coli* carrying empty pET28a as a negative control had no effect on the growth of Xoo_C2R, for which the OD_600_ value increased from 0.18 at 0 h to 0.68 at 18 h and no inhibition zone appeared. In contrast, the supernatant of LysZP protein-expressing *E. coli* culture exhibited strong inhibitory effects against Xoo_C2R, with a 1 mm wide transparent inhibition zone, and the OD_600_ remained steady from 0.18 at 0 h to 0.20 at 20 h. The good antibacterial activity of LysZP against the phage ZP3-resistant strain C2R provides a new plan for the prevention and control of Xoo strains.

## 4. Discussion

Phage therapy is commonly used to control bacterial diseases in humans [37], aquaculture [38], and agriculture [25] due to its effective and specific bactericidal ability without concerns regarding environmental pollution [39]. According to Nakayinga et al., 168 phages have been identified and applied in plant disease biocontrol caused by *Xanthomonas* species, leading to a better yield [5]. Based on the latest phage taxonomy [34], *Xanthomonas* phages can be divided into *Pradovirus*, *Bosavirus*, *Xooduovirus*, *Riverridervirus*, *Beograduvirus*, *Tsukubavirus*, *Alachuavirus*, *Carpasinavirus*, *Dibbivirus*, *Eisenstarkvirus*, *Foxquatrovirus*, *Foxunavirus*, *Klementvirus*, *Xipdecavirus*, *Coriovirus*, *Lophivirus,* and *Xylivirus*, showing the diversity of *Xanthomonas* phages.

However, the emergence of phage-resistant bacteria strains has made it necessary for the search for novel phages. Zhang et al. discovered a spontaneous phage-resistant Xoo mutant C2R, which hindered the application of phage X2 to BLB [25]. In this study, phage ZP3 was reported to have a broad host range, infecting 28 out of 56 Xoo strains, with a high lytic efficiency at MOI = 0.01. Earlier, in 1960, Wakimoto divided Xoo phages into OP1 and OP2 according to the morphological and serological properties [40]. Based on genome sequencing, ZP3 had a linear double-stranded DNA genome with a length of 44,713 bp and a G + C content of 52%, which showed 84.95% identity to the phage OP1 genome of 43,785 bp with 51% G + C content [41]. By contrast, ZP3’s genome was different from that of phage OP2 in terms of genome size (47 kb) and G + C content (61%) [42]. Phylogenetic analysis of the conservative phage terminase large subunit gene further indicated that ZP3 should be regarded as an OP1-related phage.

Infections caused by bacteria can be combated with endolysin, an enzyme produced by phages that breaks down the peptidoglycan cell wall [43]. Compared with phage therapy, endolysins have a broader host range and a relatively narrow spectrum compared to chemical antibiotics. Through molecular editing, the lytic spectrum of the recombinant endolysin can be changed [44]. In addition, no resistance to endolysins has been reported up to now [45]. The high specific bactericidal activity, low risk of bacterial resistance, and easy operability of endolysin make it a rising star in food safety, medical, and agricultural practice. The endolysin of ZP3 in this study (LysZP) is a small globular 19.5 kDa protein with a single muramidase domain at aa positions 31–163 and lacking a C-terminal cell wall binding domain, similar to the other Gram-negative phage endolysins (15–20 kDa).

The lysis cassette of most phages consists of two proteins, endolysin and holin. Holins can form holes in the cytoplasmic membrane at a genetically determined time to help endolysins cross the membrane. Unlike endolysins, more than 250 holins show low homology, diverse structures, and different pore-forming modes, but the topological structures show that they all contain at least one TMD and a highly charged C-terminal region, which can be the key for holin identification [46]. Three predicted holin-encoding genes of ZP3 were cloned and co-expressed with LysZP. Another possible holin gene (located at 22,907–23,200 bp), showing 38.89% and 39.29% amino acid similarity to the holins of *Xylella* phage Prado and *Xanthomonas* phage Xc10, respectively, was also cloned and tested. However, the expression of LysZP alone could lyse bacteria efficiently, and all four holin candidates failed to significantly improve the bacterial lysis ability of LysZP.

Interestingly, based on our results, the expression of ORF27 (HolZP) alone or with LysZP did not decrease cell viability but affected cell morphology. As shown in Figure 5B, *E. coli* cells expressing HolZP were slightly longer than wild-type cells, which had a short rod shape. When HolZP and LysZP were expressed together, morphological changes in cells were obvious, with the cells becoming extremely long (about 50 μm) even though the cell death rate was 30.97%, which was lower than cells expressing LysZP alone. The mechanism of this morphological change remains unknown, and the function of ORF27 needs to be further explored.

In fact, holin-independent cell lysis can be mediated by certain phage endolysins [16]. The *N*-terminus TMD of these endolysins directed the endolysin to the periplasm, acting normally as an SAR sequence [47]. LysZP also had a TMD region in the *N*-terminus. Live/dead cell staining, flow cytometry observations, as well as β-galactosidase activity assays with the LysZPΔTMD variant demonstrated the importance of TMD to LysZP in bacterial lysis and its potential as an SAR sequence. LysZP does not have a typical cleavable signal peptide, similar to phage P1’s endolysin, which uses the bacterial secretion system to reach the cell wall to split the peptidoglycan [17].

Since the expression of LysZP showed a good inhibitory effect on bacterial growth and induced a rapid decline in cell viability, protein purification can be conducted for future application. Even though soluble LysZP could be detected in supernatants using Western blotting, it was not sufficient for protein purification. Various induction conditions of proteins were optimized, including the induction temperature (16 °C, 20 °C, 30 °C, and 37 °C), induction time (30 min, 6 h, and 20 h), IPTG concentration (0.05 mM, 0.2 mM, and 1 mM), expression vector (pET28a and pET22b), and *E. coli* strain (*E. coli* BL21(DE3), *E. coli* BL21(DE1)pLysS, and *E. coli* (Rosetta)), but we still failed to obtain a sufficient amount of LysZP protein in a soluble supernatant after purification. This may be due to the deleterious effects of LysZP expression on the cell envelope. In fact, the lysozyme of phage phi Xo411, which showed high identity with LysZP (93.82%), expressed in *E. coli* largely formed inclusion bodies [48]. However, the LysZP total protein supernatant showed effective antibacterial effects against Xoo strains, which indicated the potential of LysZP’s application in bacterial disease control.

## 5. Conclusions

Overall, through phage morphology observation, one-step growth curve analysis, and genome sequencing, a novel Xoo phage ZP3, which belongs to the genus *Xipdecavirus,* is reported with specific infectivity and a relatively broad host range, including many Xoo strains. These characteristics indicate the potential of ZP3 as a biocontrol agent against bacterial leaf blight. Further study of ZP3’s endolysin indicated that the expression of LysZP led to cell morphological change, the destruction of cell membrane integrity, and bacterial lysis. The *N*-terminal TMD functions as an SAR sequence to mediate the exportation of LysZP to the periplasm and thus affects the lysis ability of LysZP. The LysZP protein supernatant showed antibacterial effects on Xoo strains. This study not only provided a novel Xoo phage for ecological control of bacterial leaf blight but also elucidated how an SAR-endolysin, LysZP, contributes to bacterial lysis.

## Figures and Tables

**Table 1:** Plasmids used in this study.

Plasmids	Description	Sources
pET28a	Km^R^; expression vector	Novagen (Madison, WI, USA)
pET28a-LysZP	Km^R^; recombinant expression vector with LysZP	This study
pET28a-HolZP	Km^R^; recombinant expression vector with HolZP	This study
pET28a-LysZP+HolZP	Km^R^; recombinant expression vector with LysZP and HolZP	This study
pET28a-LysZPΔTMD	Km^R^; expression vector recombinant expression vector with LysZP without TMD	This study

**Table 2:** Primers used in this study.

Primers Name	Sequences (5′-3′)	Description
Lys-F	GCAAATGGGTCGCGGATCCATGAGCGAATCCGCGAAG	Gene of Lys from ZP3
Lys-R	GGAGCTCGAATTCGGATCCACGCGTCGCCATAATAGCAG	
Hol-F	GCAAATGGGTCGCGGATCCATGTCAATGCTGCTATTATGGCG	Gene of Hol from ZP3
Hol-R	GGAGCTCGAATTCGGATCCCAGGGACGGAGGTACTGCTC	
Lys-hol-F	CTGCTATTATGGCGACGCGTATGTCAATGCTGCTATTATGGCG	Gene of Lys and Hol from ZP3
Lys-hol-R	CATAATAGCAGCATTGACATACGCGTCGCCATAATAGCAG	
Lys-TMD-F	GCAAATGGGTCGCGGATCCATGAGCGAATCCGCGAAGCAGCAGGACCATGAGGGTCTCAGGTACTC	Gene of Lys without TMD from ZP3

Restriction enzyme recognition sites are indicated by underlined nucleotides (BamHI).

**Table 3:** Host range of phage ZP3.

Strains	Sensitivity to Phage ZP3	Origin	Species	Strains	Sensitivity to Phage ZP3	Origin	Species
C2	−	Guangdong, China	Xoo	Z2	-	Zhejiang, China	Xoo
C4	−	Guangdong, China	Xoo	Z3	−	Zhejiang, China	Xoo
C8	−	Guangdong, China	Xoo	Z4	−	Zhejiang, China	Xoo
G1	+	Guangdong, China	Xoo	L1	−	Liaoning, China	Xoo
G2	+	Guangdong, China	Xoo	L2	−	Liaoning, China	Xoo
G3	+	Guangdong, China	Xoo	L3	−	Liaoning, China	Xoo
G4	+	Guangdong, China	Xoo	L4	−	Liaoning, China	Xoo
G5	+	Guangdong, China	Xoo	L5	−	Liaoning, China	Xoo
G6	+	Guangdong, China	Xoo	L6	−	Liaoning, China	Xoo
G8	+	Guangdong, China	Xoo	LN4	−	Liaoning, China	Xoo
G9	+	Guangdong, China	Xoo	LN18	−	Liaoning, China	Xoo
G10	+	Guangdong, China	Xoo	ScYc-b	+	Sichuan, China	Xoo
G11	−	Guangdong, China	Xoo	YN1	−	Yunnan, China	Xoo
G12	+	Guangdong, China	Xoo	YN7	−	Yunnan, China	Xoo
G13	+	Guangdong, China	Xoo	YN11	−	Yunnan, China	Xoo
G14	+	Guangdong, China	Xoo	YN18	−	Yunnan, China	Xoo
G15	−	Guangdong, China	Xoo	YN24	+	Yunnan, China	Xoo
G16	+	Guangdong, China	Xoo	HEN	+	Henan, China	Xoo
GD414	−	Guangdong, China	Xoo	HEN11	+	Henan, China	Xoo
Y1	−	Zhejiang, China	Xoo	FuJ	+	Fujian, China	Xoo
Y2	−	Zhejiang, China	Xoo	OS198	+	Guangxi, China	Xoo
Y3	+	Zhejiang, China	Xoo	GX1	+	Guangxi, China	Xoo
Y4	−	Zhejiang, China	Xoo	GX2	+	Guangxi, China	Xoo
Y5	−	Zhejiang, China	Xoo	GX3	+	Guangxi, China	Xoo
Y6	−	Zhejiang, China	Xoo	GX4	+	Guangxi, China	Xoo
Y7	+	Zhejiang, China	Xoo	Pxo99A	−	Philippines	Xoo
Y8	+	Zhejiang, China	Xoo	RS105	−	China	Xoc
T1	+	Zhejiang, China	Xoo	RS-1	−	Zhejiang, China	Ao
T173	+	Zhejiang, China	Xoo	RS-2	−	Zhejiang, China	Ao
Z1	−	Zhejiang, China	Xoo	R456	−	Zhejiang, China	Bs

“+” and “−“ indicate susceptible or not susceptible to phage ZP3 infection, respectively.

## Data Availability

The original contributions presented in the study are included in the article/Supplementary Materials, further inquiries can be directed to the corresponding authors.

## Supplementary Materials

The following supporting information can be downloaded at: https://www.mdpi.com/article/10.3390/v16091450/s1, Figure S1: Host range spots tests of phage ZP3; Figure S2: Induced expression and WB detection of LysZP protein; Table S1: Gene annotation analysis of the whole genome of phage ZP3.
