# Supplementary material for: Genomic Characterization of Phage ZP3 and Its Endolysin LysZP with Antimicrobial Potential against Xanthomonas oryzae pv. oryzae
Source: Viruses. 2024 Sep 11;16(9):1450. doi: 10.3390/v16091450 (PMC11437452; doi:10.3390/v16091450)
Supplement: Supplementary file 1 [file viruses-16-01450-s001.zip › viruses-3121862-supplementary.pdf]

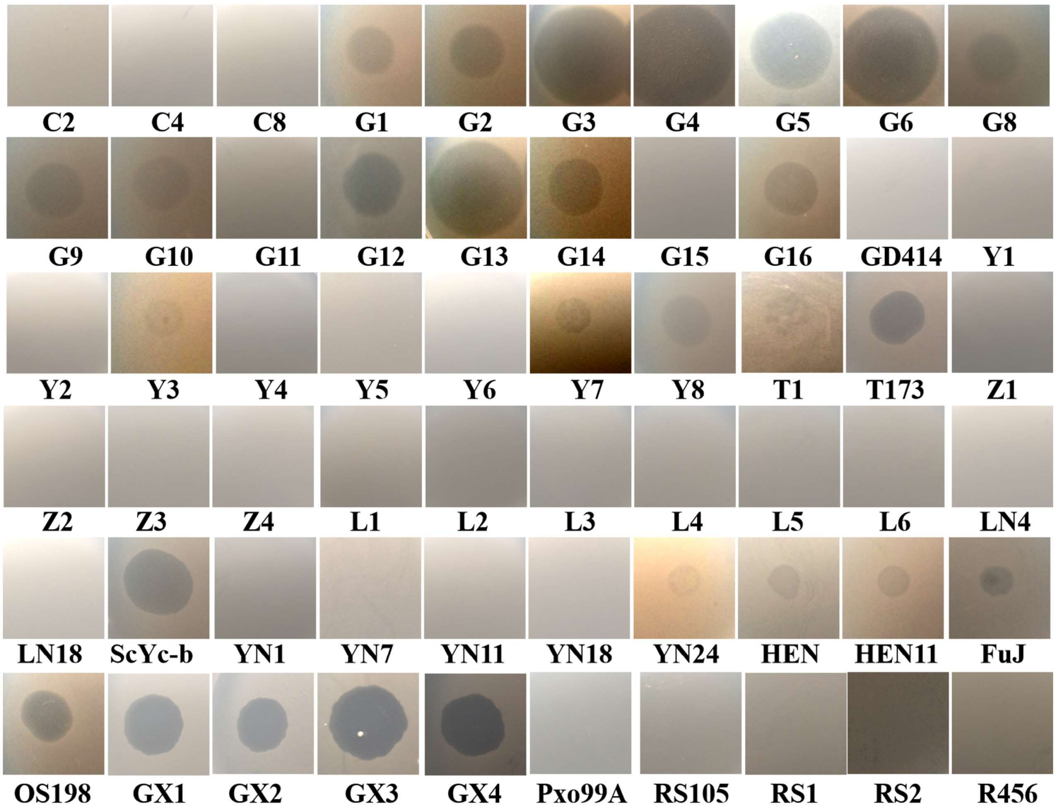

Figure S1. Host range spots tests of phage ZP3.

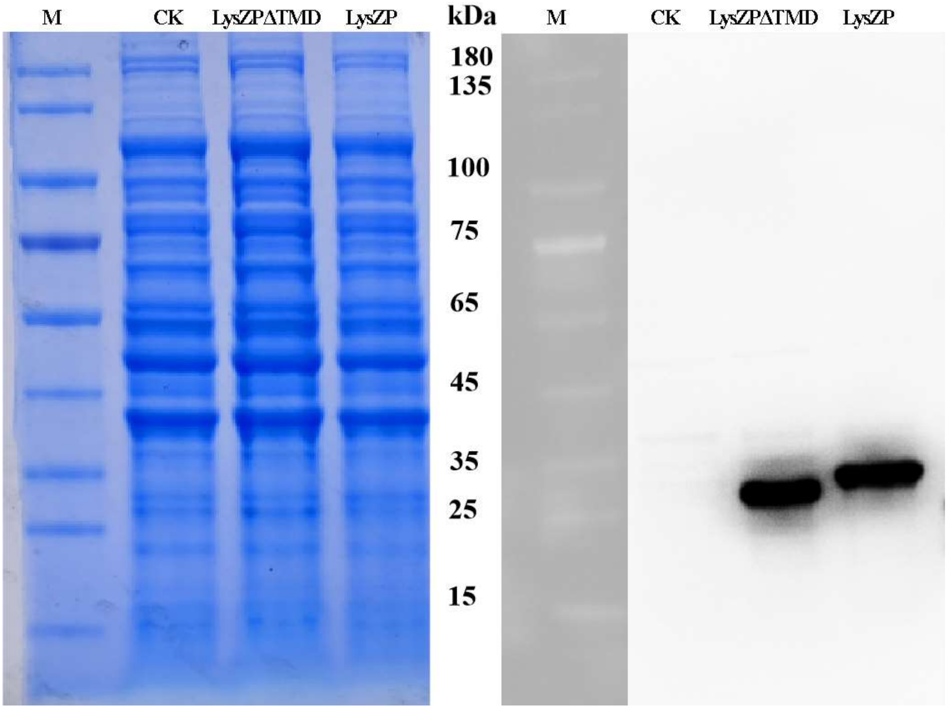

Figure S2. Induced expression and WB detection of LysZP protein.

Table S1. Gene annotation analysis of the whole genome of phage ZP3.

ORF	Coding region (bp)	Strand	Protein size (aa)	Putative function	Best match (amino acids identity)	GenBank accession no.	Signal peptide
1	112-330	+	72	1R	*Xanthomonas* phage Xop411 (91.67%)	YP_001285674.1	-
2	327-632	+	101	Hypothetical protein	*Xanthomonas* phage Xp10 (99.01%)	NP_858949.1	-
3	719-931	+	70	Phage DNA binding protein	*Xanthomonas* phage Xp10 (82.81%)	NP_858952.1	-
4	918-2603	+	561	Terminase large subunit	*Xanthomonas* phage Xp10 (97.33%)	NP_858953.1	-
5	2613-3911	+	432	Phage portal protein	*Xanthomonas* phage Xp10 (98.84%)	NP_858954.1	-
6	3920-4567	+	245	Phage head, head-tail preconnector protease C	*Xanthomonas* phage Xop411 (99.18%)	YP_001285678.1	-
7	4677-5849	+	390	Phage major capsid protein	*Xanthomonas* phage OP1 (99.23%)	YP_453565.1	-
8	5896-6243	+	115	Head-tail connector protein	*Xanthomonas* phage Xp10 (96.52%)	NP_858957.1	-
9	6243-6617	+	124	Phage protein	*Xanthomonas* phage Xop411 (98.39%)	YP_001285681.1	-
10	6607-7080	+	157	Phage capsid and scaffold	*Xanthomonas* phage OP1 (92.99%)	YP_453568.1	-
11	7077-7433	+	118	Phage protein	*Xanthomonas* phage OP1 (93.22%)	YP_453569.1	-
12	7450-8082	+	210	Tail protein	*Xanthomonas* phage OP1 (98.57%)	YP_453570.1	-
13	8086-8388	+	100	15R	*Xanthomonas* phage Xp10 (100.00%)	NP_858962.1	-
14	8783-8983	+	66	Phage protein	*Xanthomonas* phage OP1 (98.48%)	YP_453573.1	-
15	8993-10321	+	442	Phage protein	*Xanthomonas* phage OP1 (80.00%)	YP_453574.1	-
16	10365-13361	+	998	Tail length tape measure protein	*Xanthomonas* phage OP1 (94.99%)	YP_453575.1	-

ORF	Coding region (bp)	Strand	Protein size (aa)	Putative function	Best match (amino acids identity)	GenBank accession no.	Signal peptide
17	13358-13714	+	118	Phage protein	*Xanthomonas* phage L522 (97.46%)	WBK39716.1	-
18	13711-14169	+	152	Phage protein	*Xanthomonas* phage Xp10 (98.68%)	NP_858967.1	-
19	14172-14564	+	130	Phage peptidoglycan hydrolase	*Xanthomonas* phage Xp10 (97.69%)	NP_858968.1	-
20	14549-19264	+	1571	tail Component protein	*Xanthomonas* phage OP1 (97.96%)	YP_453579.1	-
21	19599-20294	+	231	Phage protein	*Xanthomonas* phage L522 (69.26%)	WBK39720.1	-
22	20291-20665	+	124	25L	*Xanthomonas oryzae* (75.00%)	WP_053520537.1	-
23	20771-21268	+	165	Phage HNH homing endonuclease	*Xanthomonas* phage Xop411 (62.42%)	YP_001285711.1	-
24	21384-22676	+	430	Deduced tail fiber protein	*Xanthomonas* phage OP1 (88.63%)	YP_453583.1	-
25	22679-22888	+	69	27R	*Xanthomonas* phage Xop411 (94.20%)	YP_001285696.1	-
26	23100-23633	+	177	Phage endolysin	*Xanthomonas* phage Xp10 (95.45%)	NP_858975.1	-
27	23608-23937	+	109	Putative holin	*Xanthomonas* phage OP1 (92.66%)	YP_453586.1	-
28	24160-24336	-	58	Phage protein	*Xanthomonas* phage L522 (87.93%)	WBK39665.1	-
29	24333-25208	-	291	DNA ligase, phage-associated	*Xanthomonas* phage Xop411 (78.35%)	YP_001285700.1	-
30	25212-25709	-	165	Phage HNH homing endonuclease	*Xanthomonas* phage Xop411 (69.14%)	YP_001285711.1	-
31	25687-25878	-	63	Hypothetical protein	*Xanthomonas* phage L522 (85.25%)	WBK39669.1	-
32	26009-28309	-	766	Phage DNA-directed RNA polymerase	*Xanthomonas* phage L522 (93.57%)	WBK39670.1	-
33	28275-28847	-	190	33L	*Xanthomonas* phage L522 (92.63%)	WBK39671.1	-

ORF	Coding region (bp)	Strand	Protein size (aa)	Putative function	Best match (amino acids identity)	GenBank accession no.	Signal peptide
34	28844-29101	-	85	Phage protein	*Xanthomonas* phage OP1 (93.90%)	YP_453593.1	-
35	29111-29950	-	279	Phage exonuclease	*Xanthomonas* phage OP1 (97.13%)	YP_453594.1	-
36	29937-30350	-	137	Phage endonuclease	*Xanthomonas* phage Xp10 (97.78%)	NP_858983.1	-
37	30347-31276	-	309	Phage exonuclease	*Xanthomonas* phage Xp10 (95.47%)	NP_858984.1	-
38	31289-32086	-	265	Phage protein	*Xanthomonas* phage L522 (98.11%)	WBK39676.1	-
39	32088-34472	-	794	DNA polymerase	*Xanthomonas* phage L522 (95.59%)	WBK39677.1	-
40	34462-35778	-	438	Phage helicase	*Xanthomonas* phage OP1 (92.69%)	YP_453599.1	-
41	35775-36617	-	280	DNA primase	*Xanthomonas* phage OP1 (86.79%)	YP_453600.1	-
42	36624-36902	-	92	Phage protein	*Xanthomonas oryzae* (81.33%)	KOR44993.1	-
43	37004-37672	-	222	Phage flagellar hook-length control protein fliK	*Xanthomonas* phage Xp10 (92.34%)	NP_858991.1	-
44	37778-38095	-	105	Hypothetical protein	*Xanthomonas* phage L522 (94.29%)	WBK39684.1	-
45	38092-38313	-	73	Phage inhibitor of transcription initiation and antiterminator	*Xanthomonas* phage Xop411 (93.15%)	YP_001285715.1	-
46	38384-38587	-	67	46L	*Xanthomonas* phage Xp10 (92.54%)	NP_858994.1	-
47	39252-39710	-	152	Phage protein	*Xanthomonas* phage L522 (70.95%)	WBK39687.1	-
48	39691-39867	-	58	HNH endonuclease family protein	*Xanthomonas* phage L522 (70.91%)	WBK39688.1	-
49	39864-40295	-	143	HslV family peptidase	*Xanthomonas* phage Xop411 (92.91%)	YP_009640111.1	-

ORF	Coding region (bp)	Strand	Protein size (aa)	Putative function	Best match (amino acids identity)	GenBank accession no.	Signal peptide
50	40323-40475	-	50	51L	*Xanthomonas* phage Xp10 (98.00%)	NP_858999.1	-
51	40754-41134	-	126	Hypothetical protein	*Xanthomonas* phage pXoo2107 (97.62%)	UUR56240.1	+
52	41133-41330	+	65	Hypothetical protein	-	-	-
53	41444-41758	-	104	Phage protein	*Xanthomonas* phage XPP1 (89.42%)	YP_010052467.1	-
54	41866-42198	-	110	53L	*Xanthomonas oryzae* (60.55%)	WP_053520478.1	+
55	42209-42418	-	69	54L	*Xanthomonas* phage Xp10 (78.18%)	NP_859002.1	-
56	42468-42632	-	54	Phage HNH homing endonuclease	*Xanthomonas* phage L522 (84.78%)	WBK39691.1	-
57	42722-42928	-	68	55L	*Xanthomonas* phage Xp10 (100.00%)	NP_859003.1	-
58	43220-43420	-	66	Hypothetical protein	*Xanthomonas* phage L522 (50.77%)	WBK39695.1	-
59	44328-44597	-	89	Hypothetical protein	-	-	-
